# Prediabetes, diabetes, and the risk of progression to diabetes among working population in Beijing-the Tongren HealthCare Study

**DOI:** 10.1371/journal.pone.0343993

**Published:** 2026-05-20

**Authors:** Yu Li, Wen Liu, Xuemei Zeng, Jing Cui, Dongning Chen, Yu Wang, Xi Cao

**Affiliations:** 1 Department of Physical Examination, Beijing Tongren Hospital, Capital Medical University, Beijing, China; 2 Department of Clinical Nutrition, Beijing Tongren Hospital, Capital Medical University, Beijing, China; 3 Center for Applied Statistics and School of Statistics, Renmin University of China, Beijing, China; 4 Department of Endocrinology, Beijing Diabetes Institute, Beijing Tongren Hospital, Capital Medical University, Beijing, China; The Chinese University of Hong Kong, HONG KONG

## Abstract

**Introduction:**

Our retrospective cohort study (3–9 years) describes the progression and regression patterns among working adults aged 18–65 with normoglycemia, prediabetes, and diabetes, providing evidence for diabetes prevention and management strategies in this population.

**Research design and methods:**

15,765 subjects received baseline examinations between 2014 and 2018, with 14,623 (92.7%) completing follow-up, representing a follow-up period of 3–9 years for analysis. Chi-square tests were used to compare prediabetes and diabetes incidence, as well as rates of glycemic normalization among different age groups and genders in working populations. Cox proportional hazard regression models were used to estimate hazard ratios (HRs) and 95% confidence interval (CI).

**Results:**

Among participants with baseline normoglycemia, 23.9% progressed to prediabetes, and 2.3% developed diabetes. Among prediabetic individuals, 28.0% progressed to diabetes, and 18.7% reverted to normoglycemia. Younger adults (18–40 years) exhibited significantly lower progression rates from normoglycemia to both prediabetes and diabetes compared to middle-aged adults (40–65 years) (prediabetes: 15.8% vs. 37.8%; diabetes: 1.2% vs. 4.2%; P < 0.001). Cox models revealed that young prediabetic individuals had a significantly higher risk of developing diabetes than middle-aged prediabetic individuals, showing a pronounced age-dependent risk pattern: prediabetic individuals aged 18–40 had an adjusted hazard ratio (HR) of 22.1 (95% CI: 14.9–32.7), compared to HR = 9.12 (7.45–11.2) in those aged 40–65.

**Conclusions:**

Prediabetes among working-age adults, particularly in younger individuals (18–40 years), carries an exceptionally elevated risk of progression to diabetes. The observed sex disparities in progression among young adults highlight the need for age- and sex-specific prevention strategies.

## 1 Introduction

Prediabetes, defined by the American Diabetes Association (ADA) as an intermediate hyperglycemic state characterized by either glycated hemoglobin (HbA1c) levels of 5.7%−6.4% or fasting plasma glucose levels of 100–125 mg/dL [1], represents a critical window for preventing type 2 diabetes mellitus (T2DM). Epidemiological studies reveal a substantial global burden, with nearly one-third of adults in both China and the United States meeting prediabetes criteria [2,3]. Approximately 5–10% of individuals with prediabetes progress to diabetes annually, and up to 70% may eventually develop the condition [4]. Insulin resistance and β-cell dysfunction are considered the two critical pathological mechanisms underlying the onset and progression of prediabetes and type 2 diabetes [4].

Notably, prediabetes remains a reversible metabolic state. The Da Qing Diabetes Prevention Study [5], the Diabetes Prevention Program (DPP) [6], and the Finnish Diabetes Prevention Study [7] have consistently demonstrated that intensive lifestyle interventions not only delay T2DM onset but also reduce cardiovascular events, microvascular complications, and all-cause mortality, ultimately improving life expectancy in this population.

A recent cohort study from the Atherosclerosis Risk in Communities (ARIC) Study of older adults (aged 71–90 years) demonstrated that during the 6.5-year follow-up period, 9% of the prediabetic population and 3% of the normoglycemic population at baseline progressed to diabetes, while 13% of the prediabetic population regressed to normoglycemia [8]. Emerging evidence also highlights prediabetes prevalence in younger populations, with cross-sectional studies reporting rates of 20% in US adolescents (12–18 years) and 25% in young adults (19–34 years) [9].

Despite these advances, critical gaps persist in understanding the progression of prediabetes in working populations (18–65 years). A high-risk demographic where early intervention could substantially mitigate future public health burdens. Current evidence predominantly focuses on elderly or pediatric populations, while progression dynamics and sex-specific risks in the working population remain poorly defined. This population’s metabolic deterioration may lead to increase incidence of diabetes-related complications, placing significant strain on healthcare systems. Our study investigates glycemic progression in a stratified working-age cohort (18–65 years), quantifying age- and sex-dependent transition rates from prediabetes to diabetes. By identifying critical windows for intervention, these findings may guide cost-effective prevention programs to curb diabetes escalation in this pre-disease state.

## 2 Materials and methods

### 2.1 Study design and participants

**Fig 1:**
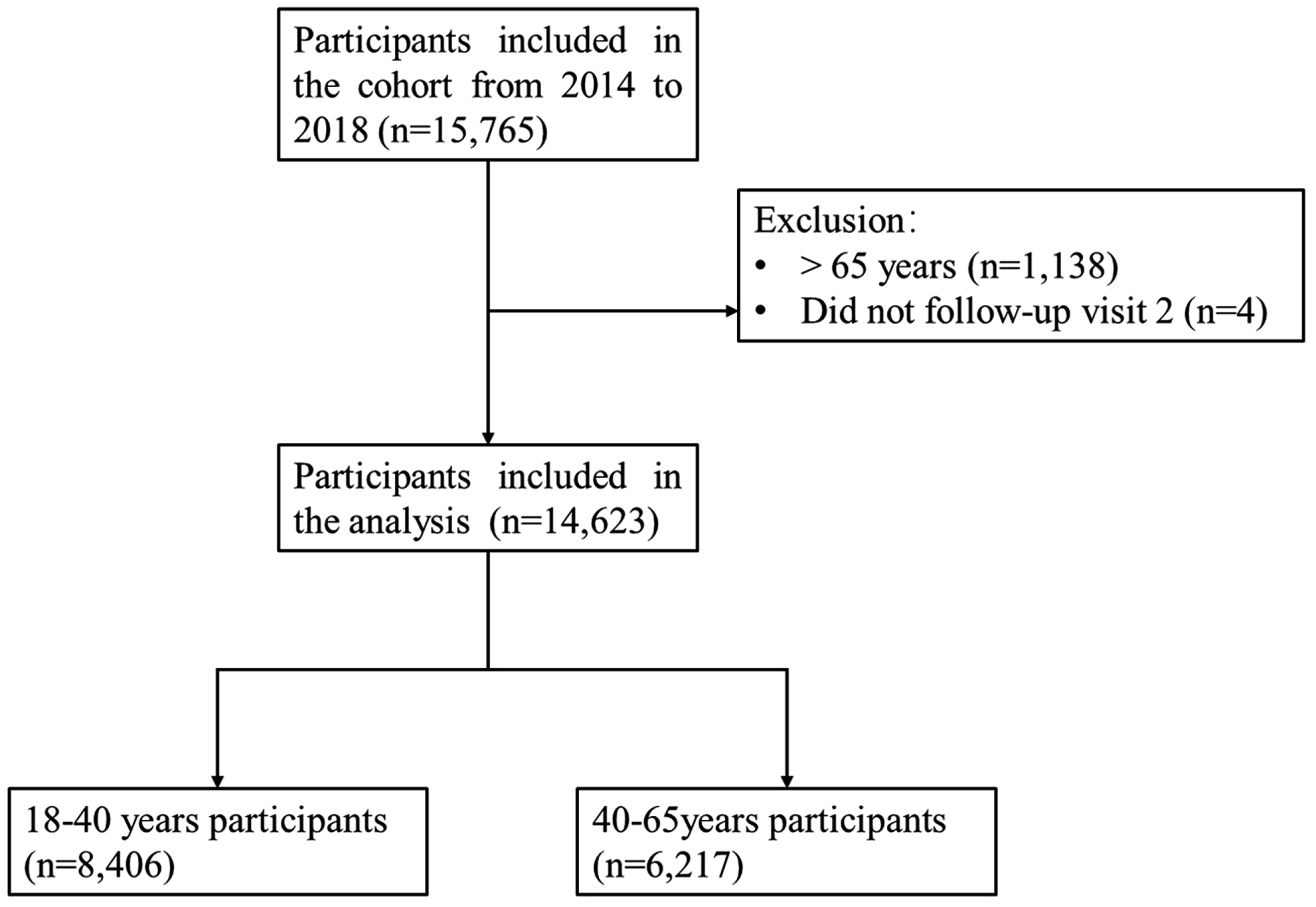
The flow chart of the study population.

This is a retrospective study, we used data from the Tongren Health Care Study, which included individuals who attended annual healthcare check-up examinations conducted in the Beijing Tongren Hospital, Affiliated with Capital Medical University. Beijing Tongren Hospital is located in Dongcheng District, a central urban area of Beijing, China. The majority of participants were affiliated with various organizations, including enterprises (34.9%), government offices (24.8%), hospitals (16.7%), academic institutions (3.6%), and religious institutions (0.7%), either as employees or retirees. These individuals had access to free healthcare through their employment [10]. We conducted the retrieval of patients’ medical records and surveyed data on December 20, 2024. The eligibility criterion for inclusion was being aged between 18–65 years. A total of 15,765 subjects received baseline examinations in the period from 2014 to 2018, and of these, 14,623 individuals participated in the follow-up assessment (Visit 2) conducted between 2018 and 2022, yielding a variable follow-up period of 3–9 years. The final analysis included 14,623 individuals (Fig 1). Ethical approval was obtained from the Medical Ethics Committee of Beijing Tongren Hospital (Approval number: TRECKY2020-066). All participants provided written informed consent before taking part in the study.

### 2.2 Data collection procedures

General demographic data, including gender and age were obtained during the first clinical visit. Blood samples were collected at baseline and visit 2 after overnight fasting to measure fasting blood glucose and HbA1c. Both FBS and HbA1c were measured twice, at baseline and during the second follow-up, respectively. The detection method has been described in detail previously [10].

### 2.3 Definition of prediabetes and diabetes

According to ADA definitions, we categorized participants for prediabetes based on fasting glucose levels of 100–125 mg/dL (5.5–6.9 mmol/L and/or HbA1c levels (5.7%−6.4%), and for diabetes based on fasting glucose level ≥125 mg/dL (≥6.9 mmol/L and/or HbA1c levels (≥6.5%).

### 2.4 Statistical analysis

Statistical analyses were performed using R software (R Foundation for Statistical Computing, version 4.4.3). Variables with normal distribution were described as means with standard deviations, while the variables of non-normal distribution were presented as medians with interquartile ranges. Between-group comparisons for continuous measures employed parametric Student’s *t*-tests or nonparametric Wilcoxon rank-sum tests based on distributional assumptions. Frequency data were displayed as counts with proportions, with group differences assessed using Pearson’s χ² tests or exact tests for small cell frequencies. To evaluate the association between baseline glycemic status and subsequent diabetes development in the working population cohort, we implemented time-to-event analyses through Cox proportional hazards modeling. These models generated hazard ratio estimates accompanied by 95% confidence limits. The proportional hazards assumption was verified using Schoenfeld residuals. For all analyses, a two-tailed *P* value <0.05 was considered to be statistically significant.

## 3 Results

### 3.1 Demographic and clinical characteristics of the study population

Among the 14,623 eligible participants, the mean age was 39 years, with 45% male, and 55% female. Among them, 8,406 were aged 18–40 years, and 6,217 were aged 40–65 years (2014–2018, baseline). According to the ADA criteria, 5% of participants had prediabetes and 4% had diabetes (Table 1).

**Table 1:** Baseline demographic and clinical characteristics of the study population, stratified by age of the working population.

Characteristic	Total	18-40 years	40-65 years
Age, mean (SE), y	39 (11.3)	31 (4.9)	50 (7.0)
**Sex**			
Male	6629 (45%)	3677 (44%)	2952 (47%)
female	7994 (55%)	4729 (56%)	3265 (53%)
Height Median	1.66 (0.13)	1.67 (0.13)	1.65 (0.13)
Weight(kg) median	65 (19)	63 (20.85)	66 (18)
BMI mean	23.62 (3.71)	22.9 (3.87)	24.3 (3.37)
FG Median (range), mmol/L	5.12 (2.99-21.14)	4.99 (2.99-20.18)	5.33 (3.38-21.14)
HbA1c Median (range), %	5.5 (3.4-13.1)	5.4 (3.4-12.0)	5.6 (3.7-13.1)
**No. (%) of** **Normoglycemia**			
HbA1c < 5.7% or FG < 100 mg/dL	12547 (86%)	7923 (94%)	4624 (74%)
**No. (%) of** **Prediabetes**			
HbA1c 5.7%−6.4% or FG 100–125 mg/dL	1448 (10%)	381 (5%)	1067 (17%)
**No. (%) of** **Diabetes**			
HbA1c≥6.5% or FG ≥ 125 mg/dL	628 (4%)	102 (1%)	526 (8%)

Abbreviation: FG. fasting blood glucose; HbA1c, glycated hemoglobin; SE, standard error.

### 3.2 Progression and regression of prediabetes and diabetes prevalence

**Fig 2:**
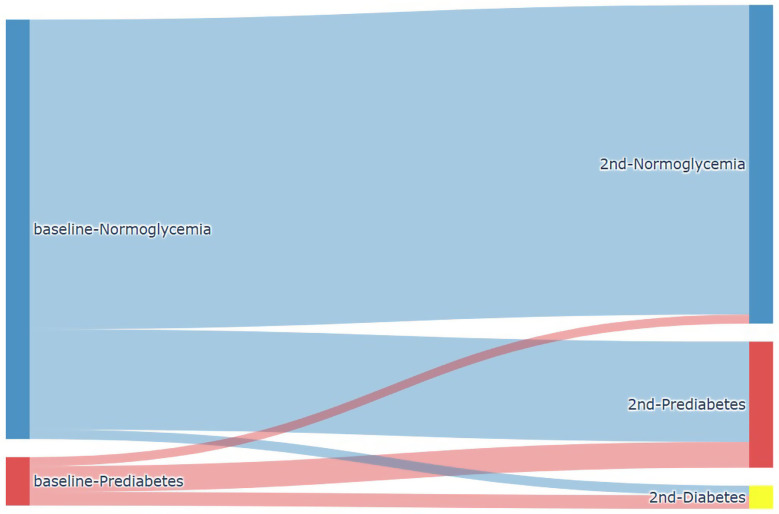
Flowchart (Sankey Plot) depicting progression and regression in working population according to normoglycemia and prediabetes at baseline.

A total of 14,623 participants attended visit 2 (2018–2022), with follow-up durations ranging from 4 to 8 years (Fig 1). Among individuals with baseline normoglycemic (HbA1c < 5.7% or FG < 100 mg/dL), 9,260 (73.8%) maintained normoglycemia at follow-up, 2,999 (23.9%) progressed to prediabetes (HbA1c 5.7%–6.5% or FG 100–125 mg/dL) and 288 (2.3%) developed diabetes (HbA1c ≥ 6.5% or FG ≥ 125 mg/dL) at follow-up. Among those with prediabetic at baseline (HbA1c 5.7%–6.5% or FG 100–125 mg/dL; baseline 2014–2018), 271 (18.7%) reverted to normoglycemia (HbA1c < 5.7% or FG < 100 mg/dL), 772 (53.3%) remained prediabetic, and 405 (28%) progressed to diabetes (Fig 2).

Analysis revealed that **normoglycemic individuals** aged **18–40 years** developed prediabetes (1,249 (15.8%)) and diabetes (94 (1.2%)) at significantly lower rates than those aged 40–65 years (1,750 (37.8%) and 194 (4.2%) respectively; *P* < 0.001) (S1 Table). The **prediabetic** population aged **18–40 years** showed fewer diabetes conversions (86 (22.6%) vs. 319 (29.9%), *P* < 0.001) and higher rates of glycemic normalization (133 (34.9%) vs. 138 (12.9%), *P* < 0.001) compared to those aged 40–65 years (S1 Table).

**Sex-stratified analysis** showed that among younger working population (**18–40 years**), females had significantly better outcomes than males. Fewer normoglycemic females progressed to prediabetes (518 (11.5%) vs. 731 (21.4%), *P* < 0.001) or diabetes (34 (0.8%) vs. 60 (1.8%), *P* < 0.001). Among those with prediabetes, more female reverted to normalization (89 (43.2%) vs. 44 (25.1%), *P* < 0.001) and fewer progressed to diabetes (31 (15.0%) vs. 55 (31.4%), *P* < 0.001) (S2 Table). However, these sex differences were not observed in the **40–65-year-old population**. Both sexes had similar rates of progression from normoglycemia to prediabetes (1,022 (39.0%) female vs. 728 (36.3%) male, *P* = 0.06) and to diabetes (102 (3.9%) female vs. 92 (4.6%) male, *P* = 0.27), and comparable outcomes among prediabetic individuals regarding both glycemic normalization (56 (11.9%) female vs. 82 (13.8%) male, *P* = 0.41) and diabetes progression (128 (27.2%) vs. 191 (32.0%), *P* = 0.10), though females were more likely to remain prediabetic (287 (60.9%) vs. 323 (54.2%), *P* = 0.03) (S3 Table).

**BMI stratification analysis** revealed significant differences in outcomes based on baseline BMI levels among the young working-age population (18–40 years). Among those with normal baseline blood glucose, the proportions progressing to prediabetes or diabetes at the second follow-up were as follows: underweight, normal BMI, overweight, and obese individuals were (37 (71.2%) vs. 1,321 (62.7%) vs. 998 (56.2%) vs. 266 (45.0%), P < 0.001). Specifically, among those with normal baseline blood glucose, the proportions progressing to prediabetes were (14 (6.3%) vs. 435 (10.7%) vs. 458 (19.8%) vs. 323 (27.2%), P < 0.001), and the proportions progressing to diabetes were (0 (0.0%) vs. 12 (0.3%) vs. 23 (1.0%) vs. 57 (4.8%), P < 0.001). Among those with baseline prediabetes, the proportions reverting to normal blood glucose at the second follow-up were (5 (55.6%) vs. 62 (47.7%) vs. 46 (35.9%) vs. 15 (14.9%), P < 0.001), while the proportions progressing to diabetes were (0 (0.0%) vs. 9 (6.9%) vs. 32 (25.0%) vs. 42 (41.6%), P < 0.001). Among those with baseline prediabetes, the proportions remaining in the prediabetes category at the second follow-up were (37 (71.2%) vs. 1,321 (62.7%) vs. 998 (56.2%) vs. 266 (45.0%), P = 0.712), indicating no significant difference between groups (S4 and S5 Tables).

### 3.3 The Hazard Ratios for incident diabetes

**Fig 3:**
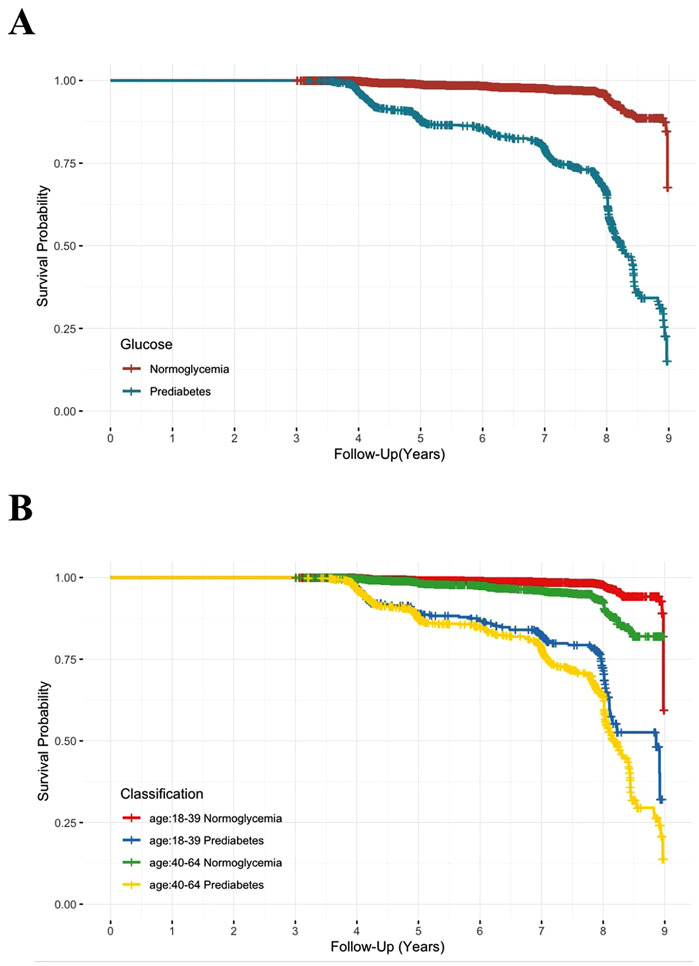
Kaplan-Meier curves depicting time-to-progression to diabetes among working-age adults (18-65 years). Figure A presents the diabetes incidence during 3-9 years of follow-up in normoglycemic (red line) versus prediabetic (blue line) individuals aged 18-65 years. Figure B demonstrates age-stratified diabetes incidence comparing normoglycemic and prediabetic groups over the same follow-up period.

Among the working-age population (18–65 years), the incidence rates of progression from normoglycemia to diabetes and from prediabetes to diabetes were 3.2 and 41.6 per 1,000 person-years, respectively. To understand the association between baseline blood glucose status and risk of developing diabetes, Cox proportional hazard models were carried out (Table 2). Compared to normoglycemia individuals, the Hazard Ratio (95%CI) of developing diabetes in prediabetic individuals aged 18–65 was 11.1 (95%CI: 9.32–13.3). When stratified by age, the HR (95%CI) was 22.1 (95%CI: 14.9–32.7) for those aged 18–40 and 9.12 (95%CI: 7.45–11.2) for those aged 40–65. Kaplan-Meier survival curves, stratified by baseline blood glucose status and age, are shown in Fig 3 AB.

**Table 2:** Incidence Rates and Adjusted Hazard Ratios (95% CIs) for Incident Diabetes, According to Prediabetes Status at Baseline in working population.

	No. of events/participants	Incidence rate per 1000 person-years (95% CI)	HR (95% CI)
**18-65 years**			
Normoglycemia	288/12547	4 (3.5-4.5)	reference
Prediabetes	405/1448	45.2 (40.9-49.9)	11.1 (9.32-13.3)
**18-40 years**			
Normoglycemia	94/7923	2.1 (1.7-2.6)	reference
Prediabetes	86/381	35.7 (28.5-44)	22.1 (14.9-32.7)
**40-65 years**			
Normoglycemia	194/4624	7.1 (6.1-8.1)	reference
Prediabetes	319/1067	48.8 (43.6-54.4)	9.12 (7.45-11.2)

## Discussion

This study analyzed longitudinal health examination data from the working population (18–65 years) in Beijing, systematically elucidating the natural history of diabetes progression among individuals with different glycemic statuses (normoglycemia, prediabetes, and diabetes) over a 3–9 years follow-up period, providing critical evidence for developing diabetes prevention strategies.

The global diabetes burden continues to escalate with a concerning trend toward younger onset [11]. Working populations exhibit significantly higher rates of overweight/obesity and dysglycemia due to chronic exposure to work-related stress, circadian disruption, unhealthy dietary patterns, and exposure to environmental pollutants [12]. Although prediabetes does not meet diagnostic criteria for diabetes, by this stage, signs of glucose intolerance and insulin resistance are often already apparent, substantially increasing risk of progression to diabetes if left unaddressed [1]. National epidemiological data reveal that in 2018, the prevalence of diabetes and prediabetes among Chinese adults aged 18–70 years was 5.0%−23.9% and 27.3%−47.6%, respectively, with surprisingly high rates in young adults aged 18–40 years (5.0%−6.5% diabetes, and 27.3%−34.2% prediabetes) [13]. Notably, our study documented significantly lower prevalence rates in this age group (1% diabetes, 5% prediabetes) compared to national data, with similar trends observed in the 40–65 age group (8% and 17%), possibly reflecting the higher socioeconomic and educational status of our study population [14].

The concept of diabetes reversibility has gained increasing empirical support. In a multi-ethnic cohort study conducted over a 4-year follow-up period, 17% of participants with prediabetes progressed to incident diabetes, whereas 36% reverted to normal glucose regulation. Furthermore, progression rates were lower among Black respondents compared to Whites, Hispanic/Latino respondents [15]. A multicenter Chinese medical examination cohort study of 15,421 individuals with prediabetes, followed for a median of 2.96 years, found that 42.03% reverted to normoglycemia, while 15.72% progressed to diabetes [16]. Our findings showed that without any intervention, conversion rates to diabetes were 1.2% and 22.6% in normoglycemic and prediabetic adults aged 18–40-year-olds, significantly lower than the 4.2% and 29.9% observed in 40–65-year-olds (specific data needed). Importantly, 34.9% of younger prediabetics reverted to normoglycemia, compared to only 12.9% in middle-aged counterparts. Multiple randomized controlled trials have demonstrated that early, intensive lifestyle interventions can significantly delay or even reverse diabetes progression [5,6]. Without intervention, approximately 7% of individuals with impaired glucose tolerance (IGT) progress to diabetes annually [17], but sustained lifestyle interventions can reduce this risk by 31%−46% over the next two decades [5]. Gender stratification revealed notable differences among individuals aged 18–40 years, females exhibited significantly lower diabetes conversion rates compared to males, from both normoglycemic (0.8% vs. 1.8%) and prediabetic (15.0% vs. 31.4%). Additionally, females demonstrated higher prediabetes reversion rates compared to males (43.2% vs. 25.1%). However, these gender specific patterns were not statistically significant in the 40–65-year age group. These findings highlight that middle-age workers, especially males represent a particularly high-risk group requiring targeted prevention strategies.

Prediabetes offers a critical opportunity for diabetes prevention. Meta-analyses indicate that Asian have a significantly higher relative risk (RR = 5.88) of progressing from prediabetes to diabetes compared to Caucasians [18]. However, our findings reveal an even greater risk, with an overall RR of 11.1 in working-age adults (18–65 years). Particularly concerning is the RR of 22.1 among young adults aged 18–40 years. This sharply contrasts with observations from elderly cohorts (45–64 years), where prediabetic individuals were more likely to revert to normoglycemia or die from other causes than to progress to diabetes [8]. These findings highlight the particularly urgent need for diabetes prevention strategies targeting working-age populations.

Our study benefits from systematic occupational health examination data with good cohort stability. However, several limitations should be acknowledged: (1) lack of serial anthropometric measurements, lifestyle data, and medication records, which limits our ability to analyze specific factors influencing glycemic changes; (2) reliance on fasting glucose along without oral glucose tolerance tests (OGTT) or postprandial measurements; (3) absence of diabetes subtype classification; and (4) limited follow-up duration, restricting insights into long-term outcomes. Future multicenter studies incorporating these parameters will provide more comprehensive evidence.

This cohort study reveals distinct age and sex disparities in diabetes progression among working adults in Beijing. Middle-aged men individuals (40–65 years), showed higher rates of progression to diabetes, while younger adult (18–40 years) and female individuals exhibited better outcomes, including higher rates of glycemic reversion. Notably, the progression risk (RR = 11.1) substantially exceeded previous reported averages in Asian population (RR = 5.88), with the highest risk observed in young adults (RR = 22.1). These findings highlight the urgent need for targeted prevention strategies, focusing on middle-aged, male, and young high-risk working populations.

Despite limitations such as single-timepoint fasting glucose measurements and the absence of lifestyle or treatment data, the study provides valuable insights for occupational health settings. Future investigations should aim to include comprehensive metabolic profiling, lifestyle indicators, and longitudinal endpoints to strengthen causal inference and inform intervention design.

## Supporting information

S1 Table Differences in the progression to prediabetes and diabetes among normoglycemic and prediabetic working-age adults across different age groups (2014–2022). (DOCX)

S2 Table Sex-specific differences in the progression to prediabetes and diabetes among working adults aged 18–40 years (2014–2022). (DOCX)

S3 Table Sex-specific differences in the progression to prediabetes and diabetes among working adults aged 40–65 years (2014–2022). (DOCX)

S4 Table BMI-specific differences in the progression to prediabetes and diabetes among working adults aged 18–40 years (2014–2022). (DOCX)

S5 Table BMI-specific differences in the progression to prediabetes and diabetes among working adults aged 40–65 years (2014–2022). (DOCX)

S6 File Raw Employee Cohort Data (anonymized). This dataset includes baseline characteristics, clinical measurements, and follow-up data of the working population cohort from the Beijing Tongren HealthCare Study. All personally identifiable information, including date of birth, has been removed to ensure participant privacy.
(ZIP)

## Abbreviations

CI: Confidence interval
FG: fasting blood glucose
HbA1c: glycated hemoglobin
HR: Hazard Ratios
SE: standard error
