# Supplementary material for: Prediabetes, diabetes, and the risk of progression to diabetes among working population in Beijing-the Tongren HealthCare Study
Source: PLoS One. 2026 May 20;21(5):e0343993. doi: 10.1371/journal.pone.0343993 (PMC13189350; doi:10.1371/journal.pone.0343993)
Supplement: S1 Table — (DOCX) [file pone.0343993.s001.docx]

**S1 Table** Differences in the progression to prediabetes and diabetes among normoglycemic and prediabetic working-age adults across different age groups (2014-2022)

| **Status at the 2th Follow-up** | **Total** | **18-40 years** | **40-65 years** | ***P*-trend** |
| --- | --- | --- | --- | --- |
| **Normoglycemia at Baseline No. (%) of participants** | | | | |
| Normoglycemia | 9,260(73.8) | 6580 (83) | 2680 (58) | <0.001 |
| Prediabetes | 2,999(23.9%) | 1249 (15.8) | 1750 (37.8) | <0.001 |
| Diabetes | 288(2.3%) | 94 (1.2) | 194 (4.2) | <0.001 |
| **Prediabetes at Baseline No. (%) of participants** | | | | |
| Normoglycemia | 271(18.7%) | 133 (34.9) | 138 (12.9) | <0.001 |
| Prediabetes | 772(53.3%) | 162 (42.5) | 610 (57.2) | <0.001 |
| Diabetes | 405(28%) | 86 (22.6) | 319 (29.9) | 0.008 |
