# Supplementary material for: Prediabetes, diabetes, and the risk of progression to diabetes among working population in Beijing-the Tongren HealthCare Study
Source: PLoS One. 2026 May 20;21(5):e0343993. doi: 10.1371/journal.pone.0343993 (PMC13189350; doi:10.1371/journal.pone.0343993)
Supplement: S2 Table — (DOCX) [file pone.0343993.s002.docx]

**S2 Table** Sex-specific differences in the progression to prediabetes and diabetes among working adults aged 18-40 years (2014-2022)

| **Status at the 2th Follow-up** | **Male** | **Female** | ***P*-trend** |
| --- | --- | --- | --- |
| **Normoglycemia at Baseline No. (%) of participants** | | | |
| Normoglycemia | 2632 (76.9) | 3948 (87.7) | <0.001 |
| Prediabetes | 731 (21.4) | 518 (11.5) | <0.001 |
| Diabetes | 60 (1.8) | 34 (0.8) | <0.001 |
| **Prediabetes at Baseline No. (%) of participants** | | | |
| Normoglycemia | 44 (25.1) | 89 (43.2) | <0.001 |
| Prediabetes | 76 (43.4) | 86 (41.7) | 0.8206 |
| Diabetes | 55 (31.4) | 31 (15.0) | <0.001 |
