# Supplementary material for: Prediabetes, diabetes, and the risk of progression to diabetes among working population in Beijing-the Tongren HealthCare Study
Source: PLoS One. 2026 May 20;21(5):e0343993. doi: 10.1371/journal.pone.0343993 (PMC13189350; doi:10.1371/journal.pone.0343993)
Supplement: S3 Table — (DOCX) [file pone.0343993.s003.docx]

**S3 Table** Sex-specific differences in the progression to prediabetes and diabetes among working adults aged 40-65 years (2014-2022)

| **Status at the 2th Follow-up** | **Male** | **Female** | ***P*-trend** |
| --- | --- | --- | --- |
| **Normoglycemia at Baseline No. (%) of participants** | | | |
| Normoglycemia | 1184 (59.1) | 1496 (57.1) | 0.1792 |
| Prediabetes | 728 (36.3) | 1022 (39.0) | 0.0646 |
| Diabetes | 92 (4.6) | 102 (3.9) | 0.2716 |
| **Prediabetes at Baseline No. (%) of participants** | | | |
| Normoglycemia | 82 (13.8) | 56 (11.9) | 0.4052 |
| Prediabetes | 323 (54.2) | 287 (60.9) | 0.0318 |
| Diabetes | 191 (32.0) | 128 (27.2) | 0.1009 |
