# Supplementary material for: Prediabetes, diabetes, and the risk of progression to diabetes among working population in Beijing-the Tongren HealthCare Study
Source: PLoS One. 2026 May 20;21(5):e0343993. doi: 10.1371/journal.pone.0343993 (PMC13189350; doi:10.1371/journal.pone.0343993)
Supplement: S4 Table — (DOCX) [file pone.0343993.s004.docx]

**S4 Table** BMI-specific differences in the progression to prediabetes and diabetes among working adults aged 18-40 years (2014-2022)

| **Status at the 2th Follow-up** | **Lean**  **(BMI<18 kg/m^2^)** | **Normal**  **(BMI>=18, <24 kg/m^2^)** | **Overweight (BMI>=24, <28 kg/m^2^)** | **Obesity (BMI>=28 kg/m^2^)** | ***P*-trend** |
| --- | --- | --- | --- | --- | --- |
| **Normoglycemia at Baseline No. (%) of participants** | | | | | |
| Normoglycemia | 208 (93.7) | 3618(89.0) | 1830(79.2) | 807(68) | <0.001 |
| Prediabetes | 14(6.3) | 435 (10.7) | 458(19.8) | 323(27.2) | <0.001 |
| Diabetes | 0 (0.0) | 12 (0.3) | 23(1.0) | 57(4.8) | <0.001 |
| **Prediabetes at Baseline No. (%) of participants** | | | | | |
| Normoglycemia | 5 (55.6) | 62 (47.7) | 46(35.9) | 15(14.9) | <0.001 |
| Prediabetes | 4(44.4) | 59 (45.4) | 50(39.1) | 44(43.6) | 0.712 |
| Diabetes | 0(0.0) | 9 (6.9) | 32(25.0) | 42(41.6) | <0.001 |
