# Supplementary material for: Prediabetes, diabetes, and the risk of progression to diabetes among working population in Beijing-the Tongren HealthCare Study
Source: PLoS One. 2026 May 20;21(5):e0343993. doi: 10.1371/journal.pone.0343993 (PMC13189350; doi:10.1371/journal.pone.0343993)
Supplement: S5 Table — (DOCX) [file pone.0343993.s005.docx]

**S5 Table** BMI-specific differences in the progression to prediabetes and diabetes among working adults aged 40-65 years (2014-2022)

| **Status at the 2th Follow-up** | **Lean**  **(BMI<18 kg/m^2^)** | **Normal**  **(BMI>=18, <24 kg/m^2^)** | **Overweight (BMI>=24, <28 kg/m^2^)** | **Obesity (BMI>=28 kg/m^2^)** | ***P*-trend** |
| --- | --- | --- | --- | --- | --- |
| **Normoglycemia at Baseline No. (%) of participants** | | | | | |
| Normoglycemia | 37 (71.2) | 1321(62.7) | 998(56.2) | 266(45.0) | <0.001 |
| Prediabetes | 13(25.0) | 737 (35.0) | 699(39.4) | 263(44.5) | <0.001 |
| Diabetes | 2 (3.8) | 49 (2.3) | 79(4.4) | 62(10.5) | <0.001 |
| **Prediabetes at Baseline No. (%) of participants** | | | | | |
| Normoglycemia | 1 (14.3) | 56 (16.2) | 52(11.5) | 25(10.6) | 0.039 |
| Prediabetes | 5(71.4) | 231 (66.8) | 243(53.8) | 117(49.8) | <0.001 |
| Diabetes | 1(14.3) | 59 (17.1) | 157(34.7) | 93(39.6) | <0.001 |
